# Exception to the Rules?: Investigating Predictors of Late-Life Volunteerism in North Dakota

**DOI:** 10.1093/geroni/igaf122.4111

**Published:** 2025-12-31

**Authors:** Bryce Van Vleet, Emily Kinkade

**Affiliations:** Eastern Mennonite University, Harrisonburg, Virginia, United States; NDSU Extension, Fargo, North Dakota, United States

## Abstract

Volunteering in late life is predicted by younger age, higher education, and better health among other factors. Despite North Dakota being an aging, rural state with health outcomes and educational attainment lower than the national average, late-life volunteerism outpaces the US national average. The current project investigates predictors of volunteerism in North Dakotan older adults and compares those predictors to control states with similarly high rates of volunteerism, and Nevada, the state with the lowest rate of volunteerism. An older adult (65+) subset of the 2023 Current Population Survey, Volunteering and Civic Life Supplement was utilized. A logistic regression found gender, higher education, and contact with others to be significant predictors of volunteerism in North Dakota, Nebraska, and Nevada. Next, t-tests identified differences in volunteerism predictors between North Dakota and Nevada. Older North Dakotans had less contact with others, were less racially diverse, and were more rural. Last, an ANOVA was conducted to investigate differences in predictors between North Dakota and Nevada, and two regional control states: Utah and Nebraska. North Dakotans were more rural than all other comparison states, had lower education and less contact with others than Nevada only, but had greater contact with others than Utah. These findings indicate that factors other than established demographic predictors of volunteerism, such as contact with others, may be important predictors of volunteerism among older adults. Due to the exploratory nature of this study, future research is needed to investigate these implications further.

